# On the Way to the Marathon—Motivation for Participating in Mass Running Events Among Children and Adolescents: Results of the Poznan Half Marathon Pilot Study

**DOI:** 10.3390/ijerph17145098

**Published:** 2020-07-15

**Authors:** Ewa Malchrowicz-Mośko, Arkaitz Castañeda-Babarro, Patxi León Guereño

**Affiliations:** 1 Faculty of Sport Sciences, Eugeniusz Piasecki University of Physical Education, 61-871 Poznan, Poland; 2 Faculty of Psychology and Education, University of Deusto, 5001 San Sebastian, Spain; arkaitz.castaneda@deusto.es (A.C.-B.); patxi.leon@deusto.es (P.L.G.)

**Keywords:** motivation, sporting events, children, adolescents, sport management, health promotion, public health

## Abstract

The market for mass sports events geared towards adults is already saturated, while more new initiatives are exclusively targeting children and adolescents. Mass sports events for amateur athletes can be a great way to encourage young people to exercise regularly, such as in cases where physical education classes do not fully play this role. A lot of research has already been conducted on the subject of adult motivation for participating in amateur sports events, i.e., marathons, ultramarathons, duathlons or triathlons. However, the research niche is children and adolescents’ motivation. The aim of this study is to recognize motivation among children aged 12 for participating in children’s running events in Poland. The study was conducted via a diagnostic survey using the Motives for Physical Activity Measure–Revised (MPAM-R) scale to determine what motivation is most important for young athletes and whether there are any differences in terms of gender. The highest values were obtained by motivation related to fun and enjoyment during sporting events, while the lowest values were obtained by motivation related to social affiliation. Aspects associated with good fun should be promoted during activities related to the marketing of sporting events for young people. This article contains the results obtained from the Poznan Half Marathon pilot study and recommendations for future lines of research. Such results will allow us to understand the motivation behind modern young amateur athletes and to better manage mass sports events that target children and adolescents.

## 1. Introduction

We are observing the dynamic development of mass sports events around the world today. Changes occur not only on a quantitative, but also on a qualitative level; many events or disciplines are modified and modernized to attract as many people as possible. The market for mass sports events geared to adults is already saturated, while more and more initiatives are exclusively targeting children and adolescents. Mass amateur sports events can be a great way to encourage young people to exercise regularly, such as in cases where physical education classes do not fully play this role.

The transition from kindergarten to school is especially associated with a variety of negative changes—After entry to elementary school, physical activity levels decrease. Moreover, physical fitness levels of children and adolescents over the past few decades have rapidly declined; young people are spending an increasing amount of time in environments that require being constantly seated [1]. The traditional concept of education and school settings significantly contributes to the sedentary behavior of adolescents at secondary schools [2].

A lot of research has already been conducted on the subject of adult motivation for participating in amateur sports events, i.e., marathons, half marathons, ultramarathons, bicycle races or triathlons [3,4,5]. However, the research niche is children and adolescents’ motivation. Knowing that children and adolescents’ physical activity should be increased is not enough to enhance their activity frequency, intensity and duration [6].

Mummery and Brown pointed out that there is still a great deal to be learned about community physical activity interventions that engage and result in a population’s behavioral change [7], and according to Green, in order to achieve mass sports participation at a grassroots level, there needs to be a greater understanding of how to manage sports as a means for promoting active lifestyles for children and adolescents [8]. Evidence suggests that social support impacts on participation in sport or physical activity, and is associated with health benefits, although the link is complex and not well understood [9]. Understanding boys’ and girls’ motivation is important not only to attract them to sporting events, but above all to make them return to them in the future and thus to ensure they become permanently interested in physical activity and sport. Research suggests that active participation in sport in primary school affects the future motivational structure of teenagers [10]. Pannekoek et al. underlined the fact that specific motivation for physical activity in children below the age of 12 years is a largely underrepresented issue in contemporary research [11]. Although engagement in sufficient physical activity is highly important for children’s current and later health, relatively little is known about the factors that motivate children and adolescents to be physically active.

Children and adolescents’ motives for participating in sport and physical activity are complex and multi-dimensional [12]. Martin et al. showed that children are very “loyal” participants of sporting events; in the triathlon they analyzed in Australia, children, despite their young age, participated in the event up to seven times [13]. Therefore, their research results prove a strong relationship between the early age of children and their continuous participation in the event. Green et al. emphasize the special role of parental involvement in encouraging young people to participate in sport [14]. Green et al. believe that among the representatives of the social middle class, not only economic capital, enabling children to participate in many sports, but also transferred socio-cultural capital in developing a passion for sports, plays an important role in sports participation [14]. Undoubtedly, an important task for organizations responsible for the promotion of mass sports events and health promotion is certainly to learn about the motivation behind children and adolescents coming from families representing lower social classes, as well as to make events available to them. Studies by Bengoechea and Strean have shown that teenagers aged 13–17 are very sensitive to the influence of other people (like parents, trainers, peers etc.) on motivation for participating in competitive sport [15]. According to Owen et al., external regulation has a weak negative association with physical activity in children and adolescents [16]. In another study, quality of friendship had a weak relationship with self-worth and a commitment to sports among girls aged 11–14 [17]. According to Weiss, intervention strategies for maintaining and enhancing physical activity motivation in children and adolescents should include providing optimal challenges, creating a mastery motivational climate, making physical activity fun, and helping children help themselves [18].

The aim of this study is to recognize the motivation of girls and boys aged 12 for participating in mass running events.

## 2. Materials and Methods

### 2.1. Methods

The study was carried out using the diagnostic survey method. The Motives for Physical Activity Measure–Revised (MPAM-R) scale was used in the study. The MPAM-R addresses people’s motives for doing sport and contains 30 items. The following motives are evaluated: enjoyment, fitness, competence, social, and appearance. Fitness refers to being physically healthy/active, energetic, and strong. Appearance refers to becoming physically more attractive, looking better, and reaching or maintaining a desired weight. Challenge/competence refers to being physically active because of the need to improve at an activity, acquiring new skills, and facing a challenge. Social refers to being with friends and meeting new people during a sporting activity. Enjoyment refers to being physically active simply because it is interesting, fun, enjoyable, and makes us happy. The MPAM-R is a revised version of an earlier scale that went by the same name. The previous measure by Frederick and Ryan [19] was shorter, while the longer version was later validated and presented by Ryan, Frederick, Lepes, Rubio, and Sheldon [20]. The MPAM-R enables motives to be compared between different sports. Answers to items on the questionnaire are on a seven-point Likert-type scale, where 1 means not a reason and 7 represents the most important reason. Respondents did not report any problems with understanding the selected MPAM-R scale. We decided not to use the Motivations of Marathoners Scale (MOMS) questionnaire [21], which was designed specifically to study runners’ motivation, because we found it too difficult to understand and too complicated for children and adolescents.

### 2.2. Participants

The case study of this pilot research is a children’s run known as “On the way to the marathon” accompanying the Poznan Half Marathon event. This run is dedicated to children aged 12 and below, and they run a distance of 1.2 km. A total of 48 young runners aged 12 participated in our study: 28 girls (58.33%) and 20 boys (41.66%). Thirty-five children (72.91%) registered to participate in the event because it was their own decision, while 13 (27.08%) were persuaded by other people to do so (parents, peers or teachers).

### 2.3. Ethical Issues

Permission was obtained to conduct the study from the event organizers in January 2020, who provided the parents of the athletes with the online survey. According to the National Science Center in Poland, anonymous diagnostic surveys in Poland among people aged 12 and over do not require the consent of the bioethics committee. The study was in line with the Declaration of Helsinki regarding voluntary agreement for participation in diagnostic surveys. The online survey was anonymous, voluntary and confidential. Respondents—Children and parents—Were informed about the nature of the survey. We wanted children to complete the questionnaire on their own, only under parents’ supervision but without their help, i.e., in the case of studies, for example, by Martin et al. [12]. As a result, we hope that parents have not unduly impacted on responses.

### 2.4. Data Collection

We collected data from respondents from February 2020 to March 2020. Some children volunteered themselves and some were reported by schools as starting in the competition. In February and at the beginning of March 2020, the organizers of the event handed over the survey to parents and participants who registered to participate in the children’s race, which was to take place in April 2020. Coinciding with the start of the coronavirus pandemic, the Poznan Half Marathon and “On the way to the marathon” children’s run were cancelled and moved to 2021, and applications were suspended. In races organized by the same organizer, about 400 children and adolescents were left standing on the starting line. The epidemiological situation meant that the study should be treated as a pilot, illustrating certain trends in children’s behavior.

### 2.5. Data Analysis

Comparisons between means on 5 motive scales were made using the t test for independent variables, while in the event of failure to meet the assumption of equal variance (tested by Levene’s test). the Cochran–Cox test was used. The analysis of the relationship between gender and making the decision to start in the event was performed using the Chi-square test. The effect size for the t test was measured using Cohen’s d factor, while the fi factor was used for the Chi-square test. Significant results were assumed for *p* < 0.05, and calculations were made using Statistica 10.0 (Statsoft, Inc., 2011, Cracow, Poland).

## 3. Results

**Figure 1:**
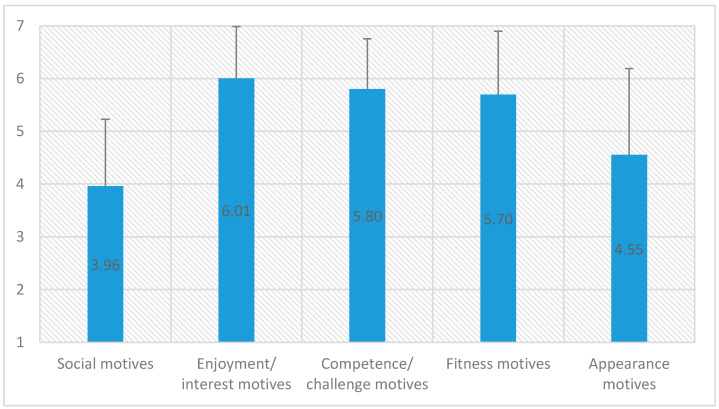
Motives of the children being surveyed.

In the first step, it was ascertained which motives were the most important for all the children being surveyed. The test results have been presented below (Table 1, Figure 1).

It turned out that the most important motives (6.01) for participating in the mass run among children were enjoyment/interest motives (e.g., it’s fun, I like doing this activity, it makes me happy) and the least important (3.96) were social motives (e.g., I want to be with my friends, I want to meet new people). The assessment of all MPAM-R motives described by respondents is shown below (1–5: social motives, 6–12: enjoyment/interest motives, 13–19: competence/challenge motives, 20–24: fitness motives, 25–30: appearance motives).

Assessment of detailed MPAM-R motives by respondents have been presented in Table 2.

**Figure 2:**
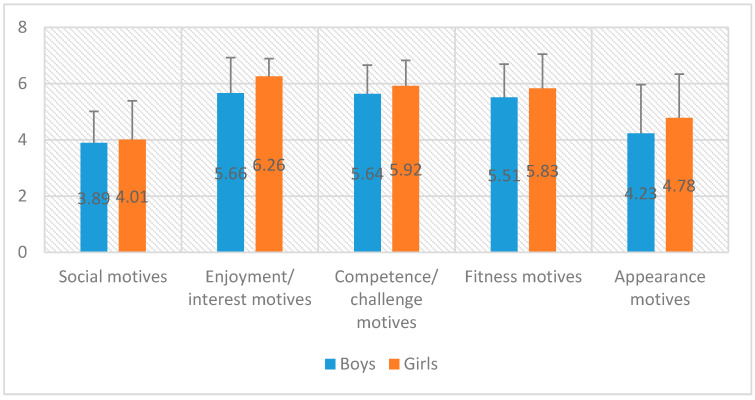
Differences in motivation depending on gender.

In the next step, we ascertained whether there were any differences in motivation depending on gender. The test results have been presented below (Table 3, Figure 2).

The result turned out to be close to the limit of the level of statistical tendenc, in the case of the enjoyment/interest motives scale (higher for girls). The groups analyzed were small, and so although the result was not significant, it represented a relatively high (albeit average) value of the effect strength.

It was also decided to ascertain the motivation for children who decided to participate in the run themselves and at the instigation of other people (Table 4).

The differences were greatest in the case of the enjoyment/interest motives scale and also the effect strength (in the “it was my decision” group). However, small numbers had a strong impact on results. In this case, the assumption of equal variance was unfulfilled and the test with correction (Cochran–Cox test) had to be used, which resulted in an increase in the probability value of *p* (calculated by the usual t test, which gave a result close to the limit).

The motivation in these two groups of respondents depending on gender was also ascertained (Table 5).

Again, the result was close to the significance limit. There is a tendency to make more frequent decisions at the instigation of others in the boys’ group. Effect strength was measured, with fi = 0.24 proving to be a small effect.

Respondents were also asked whether mass running events encouraged them to run every day. Respondents rated this impact on average at 5.5 on a seven-point Likert scale.

## 4. Discussion

It turned out that the highest values were obtained by motivation related to enjoyment/interests (6.01) during sporting events (e.g., it’s fun, I like doing this activity, it makes me happy), while the lowest (3.96) values were obtained by motivation related to social motives (e.g., I want to be with my friends, I want to meet new people). Aspects associated with good fun should be especially promoted during activities related to the marketing of sporting events for young people. Our study into gender differences in motivations shows that the result turned out to be close to the limit at the level of statistical tendency in the case of the enjoyment/interest motives scale, which was higher for girls.

Previous studies into the physical activity of children and adolescents underlined the significance of fun in sport. According to Visek et al., children cite fun as the primary reason for participation in sport and its absence as the number one reason for youth sport attrition [22]. Based on qualitative research—In-depth interviews with children through parents—Scanlan et al. found that children participate in triathlons due to factors such as competition, challenge, fun, prizes, or at the instigation of parents or friends [23]. However, in our study, competence/challenge was ranked in second position after enjoyment. Additionally, Allender et al. claimed that children are motivated mainly by enjoyment [24]. According to Cope et al., children’s participation in sport is motivated by five primary factors: perception of competence, fun and enjoyment, parents, learning new skills, and friends [25]. However, in our study, the social motives proved to be the least important (although social motives were important for adult runners in previous studies, e.g., [5,26]). The findings from studies into sports motivation among 11–15-year-old school children in Scandinavia suggest that social, fun and health motives are perceived as being the main motivation for sports participation. Competition and achievements were not rated as important reasons for liking sport, with older pupils seeming to attach more importance to sport being fun. Winning and achieving better skills in sport seem to decrease in importance as pupils grow older [27].

In our study into running, the social motives turned out to be the least important, while in the case of martial arts (judo), young girls emphasized the importance of making friends through sport in the course of the research [28].

We also decided to ascertain the motivation for children who decided to participate in the run themselves and at the instigation of other people. The differences were greatest in the case of the enjoyment/interest motives scale and also the effect strength in the “it was my decision” group. According to Lisowski et al., boys at school are more likely than girls to undertake running, cycling and team sports, while girls are more likely than boys to undertake roller skating and dancing [1]. In our study, we observed a tendency towards making more frequent decisions at the instigation of others in the boys’ group. Conversely, girls more often decided on their own about participation in the running event.

Respondents were also asked whether mass running events encouraged them to run every day. Respondents rated this impact on average at 5.5 on a seven-point Likert scale, and so this is a very positive observation in the field of sports promotion.

### 4.1. Strengths and Limitations

Children and young people, just like older people, are the most difficult to study (the least available) research group in running, and so the strength of this research focuses on the fact that it concentrates on young 12-year-old athletes. However, the results obtained from our pilot study and trends observed should be treated with caution—The group of respondents is small because the study was discontinued for unforeseeable reasons (coronavirus pandemic).

### 4.2. Future Lines of Research

We intend to conduct research on a larger group of respondents in the future, and we also encourage other researchers to do the same. It is necessary to examine the motivation of children and teenagers for participating not only in running events that have not been tested so far, but also in duathlons or triathlons. Research should be carried out according to groups of teenagers (over 12 years) and children (under 12 years) as Sallis et al. noted different sports motives for age and gender findings among children and adolescents [29]. We should also compare children and adolescents’ motivation in different types of individual and group sports.

## 5. Conclusions

In conclusion, this study shows that young athletes’ motivation for taking part in running events are related to their fun and enjoyment rather than social motives. Girls showed higher values than boys in this enjoyment dimension, although no significant differences were found gender-wise in any of the motives for participating. To conclude, we can say that mass running events encourage young runners to run every day in a fun way. Taking these conclusions into account, it seems clear that fun should be promoted during activities related to the marketing of sporting events for young people, while at the same time, promoting these events will encourage young people to run.

## Figures and Tables

**Table 1:** Motives of the surveyed children. MPAM-R: Motives for Physical Activity Measure–Revised.

MPAM-R Scale	Mean	Min	Max	Standard Deviation
Social motives	3.96	1.60	6.20	1.27
Enjoyment/interest motives	6.01	2.57	7.00	0.98
Competence/challenge motives	5.80	3.29	7.00	0.95
Fitness motives	5.70	2.20	7.00	1.20
Appearance motives	4.55	1.00	7.00	1.64

**Table 2:** Assessment of detailed MPAM-R motives.

Motives—MPAM-R Scale	Median (M)	Minimum	Maximum	Standard Deviation
1. I like to be with others who are interested in this sporting activity	3.98	1.00	7.00	1.951427
2. I want to be with my friends	4.60	1.00	7.00	1.607689
3. I want to meet new people	3.92	1.00	7.00	1.748353
4. My friends want me to take part	2.62	1.00	7.00	1.863707
5. I enjoy spending time doing this sporting activity with other people	4.67	1.00	7.00	1.860374
6. I like the excitement of participation	6.23	1.00	7.00	1.308557
7. It’s fun	6.23	2.00	7.00	1.224564
8. I like doing this activity	6.20	2.00	7.00	1.030561
9. It makes me happy	6.04	3.00	7.00	1.009705
10. I find this activity stimulating	6.17	2.00	7.00	1.260418
11. I think it’s interesting	5.39	2.00	7.00	1.364280
12. I enjoy this activity	5.77	1.00	7.00	1.574255
13. I like engaging in physically challenging activities	5.89	1.00	7.00	1.258834
14. I want to acquire new skills	5.39	1.00	7.00	1.540096
15. I want to improve my existing skills	5.96	3.00	7.00	1.090741
16. I like the challenge	5.79	2.00	7.00	1.383156
17. I want to maintain my current level of skill	5.67	1.00	7.00	1.388913
18. I like activities which are physically challenging	5.96	2.00	7.00	1.30
19. I want to get better at my activity	5.94	3.00	7.00	1.21
20. I want to be physically fit	6.31	3.00	7.00	1.01
21. I want to have more energy	5.94	1.00	7.00	1.33
22. I want to maintain my well-being and physical health	5.00	1.00	7.00	1.75
23. I want to improve my cardiovascular fitness	5.62	1.00	7.00	1.45
24. I want to maintain my physical strength in order to live a healthy lifestyle	5.60	1.00	7.00	1.47
25. I want to maintain my weight so I look better	4.77	1.00	7.00	2.13
26. I want to look attractive to others	5.48	1.00	7.00	1.58
27. I want to improve my appearance	5.02	1.00	7.00	1.88
28. I feel physically unattractive if I don’t	4.21	1.00	7.00	2.12
29. I want to improve my body shape	4.85	1.00	7.00	1.91
30. I want to define my muscles so that I look better	2.98	1.00	7.00	2.01

Notes: 1–5: social motives, 6–12: enjoyment/interest motives, 13–19: competence/challenge motives, 20–24: fitness motives, 25–30: appearance motives.

**Table 3:** Differences in motivation depending on gender. SD: standard deviation.

MPAM-R Scale	Boys (*n* = 20)		Girls (*n* = 28)		*t*	*p*	*d*
	M	SD	M	SD			
Social motives	3.89	1.12	4.01	1.38	−0.31	0.756	0.09
Enjoyment/interest motives	5.66	1.26	6.26	0.63	−1.95	0.062	0.63
Competence/challenge motives	5.64	1.02	5.92	0.90	−1.01	0.316	0.29
Fitness motives	5.51	1.18	5.83	1.22	−0.90	0.371	0.27
Appearance motives	4.23	1.73	4.78	1.56	−1.14	0.259	0.33

**Table 4:** Motives of children who decided to start in the run themselves and at the instigation of Other people.

MPAM-R Scale	Instigation of Other People (*n* = 13)		Own Decision (*n* = 35)		*t*	*p*	*d*
	M	SD	M	SD			
Social motives	3.97	1.27	3.95	1.29	0.04	0.972	0.01
Enjoyment/interest motives	5.57	1.51	6.17	0.66	−1.38	0.190	0.55
Competence/challenge motives	5.81	1.28	5.80	0.82	0.06	0.956	0.02
Fitness motives	5.48	1.47	5.78	1.10	−0.77	0.448	0.23
Appearance motives	4.81	1.76	4.46	1.61	0.65	0.516	0.21

**Table 5:** Decision about start in the run by gender.

Decision about Participation	Boys *n* = 20		Girls *n* = 28		All Together	
	*n*	%	*n*	%	*n*	%
External (parents, peers, teachers)	8	40.00%	5	17.86%	13	27.08%
Self-decision	12	60.00%	23	82.14%	35	72.92%
Altogether	20	100.00%	28	100.00%	48	100.00%
Result of Chi-square test	χ² = 2.90; df = 1; *p* = 0.089; fi = 0.24					
